# Research on edge-control methods in CNC polishing

**DOI:** 10.1186/s41476-017-0053-9

**Published:** 2017-09-16

**Authors:** Guoyu Yu, David Walker, Hongyu Li, Xiao Zheng, Anthony Beaucamp

**Affiliations:** 1 National Facility for Ultra Precision Surfaces, OpTIC Centre, University of Huddersfield, St. Asaph Business Park, Ffordd William Morgan, North Wales, St Asaph, LL17 0JD UK; 2 Department of Physics and Astronomy, University College, Gower St, London, WC1E 6BT UK; 3 Zeeko Ltd, 4 Vulcan Court, Vulcan Way, Coalville, Leicestershire, LE67 3FW UK; 4 Researh Center for Space Optical Engineering, Harbin Institute of Technology, Harbin, 150001 China; 5 Kyoto University, C3 Bldg. Kyotodaigaku-Katsura, Nishikyo-ku, Kyoto, 615-8540 Japan

**Keywords:** Segment mirror, Optical fabrication, Telescopes, Polishing

## Abstract

**Background:**

We have developed edge-control for the Precessions TM process suitable for fast fabrication of large mirror segments, and other applications sensitive to edge mis-figure. This has been applied to processing of European extremely large telescope (E-ELT) prototype mirror-segments, meeting the specification on maximum edge mis-figure. However we have observed residuals that have proved impossible to correct with this approach, being in part the legacy of asymmetries in the input edge-profiles.

**Methods:**

We have therefore compared different proposed methods experimentally and theoretically and report here on a new edge-rectification step, which operates locally on edges, does not disturb the completed bulk area.

**Results:**

A new toolpath has been developed and experiments have been carried out to demonstrate that local edge rectification can be carried out.

**Conclusions:**

With this method, the residue error on edges can be removed separately and has potential to reduce total process time.

## Background

Segmented mirrors were adopted for 10 m–class telescopes and are being extended for the forthcoming 30-40 m class [1, 2]. This concept has found applications in other areas [3]. One important requirement of mirror segments is achieving adequate control of edge mis-figure, as this can deflect stray light or infrared emissivity into the science beam, reducing contrast and signal-to-noise ratio [4]. For polishing in the semiconductor sector it can be important because of depth-of-focus limitations of photolithography, and the need to maximize silicon useful real-estate. There are published theoretical reports on modelling edge-effects in polishing, one studying the parametric tool influence functions [5]. Other reports [6, 7] considered a “skin model” related to the Preston equation. Further reports [8, 9] have presented experimental data showing variation of tool influence functions when encroaching an edge, delivering improved edge performance.

**Fig. 1:**
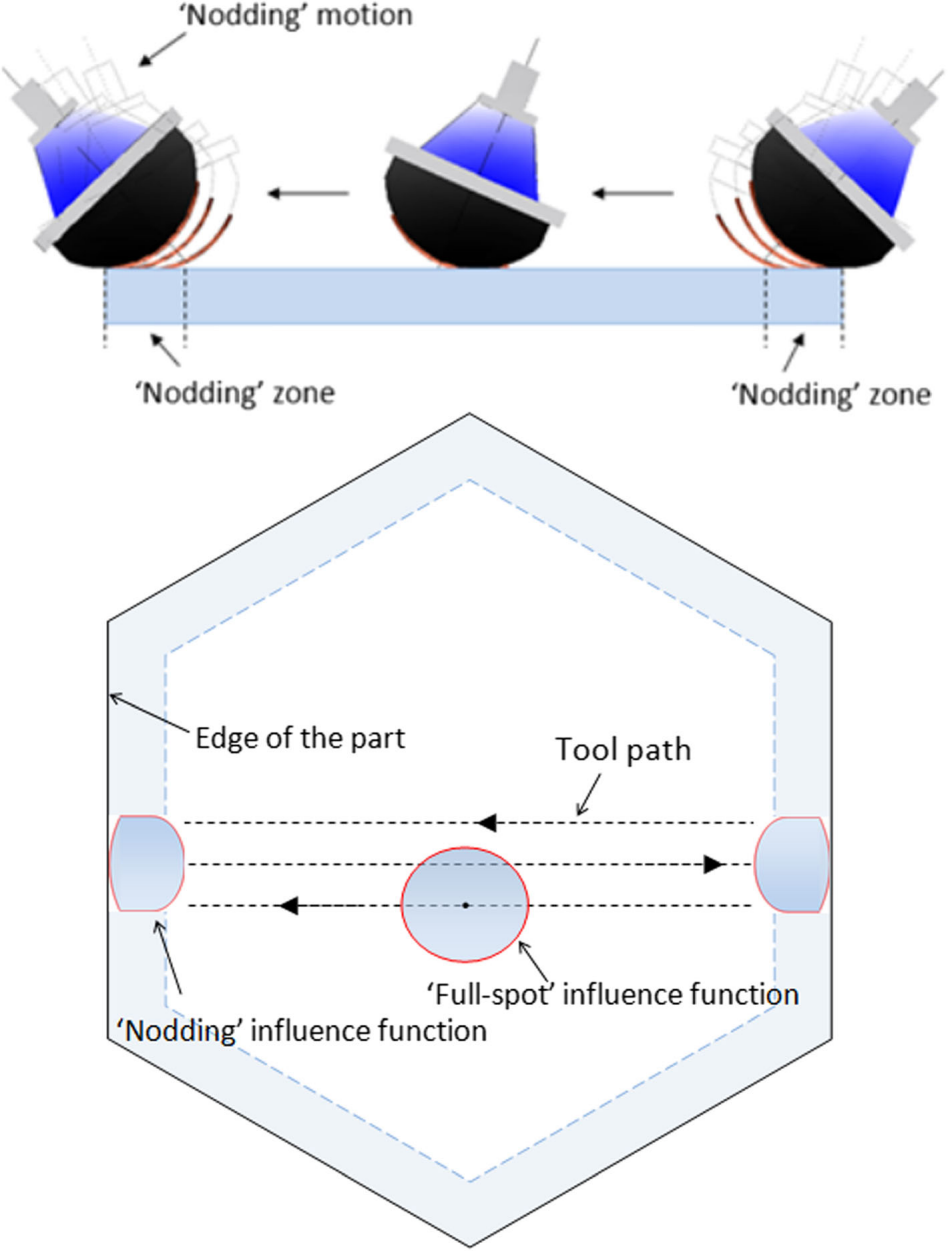
Schematic diagram showing working principle of a nodding tool (upper) and deployment of influence functions (IF) on a surface (lower)

The specified maximum edge mis-figure per edge on each E-ELT prototype segment is 200 nm PVq surface, and the average per edge over the prototypes is 100 nm. We have described in a previous paper [10] an end-to-end process-chain for mirror segments, targeted at the E-ELT, as well as other applications such as semiconductor polishing. This work has provided evidence that a fast, cost-effective process for polishing of the bulk surface and edges, directly on precision-ground aspheric hexagons, is achievable. This is based on bonnet-polishing of the entire segment with a raster tool-path (Fig. 1). The tool-lift algorithm progressively reduces spot-size of the near-Gaussian influence function, or ‘IF’) towards the outer extreme of the edge-zone (which we define as the peripheral zone one full-spot-size wide). This leaves a controllable edge up-stand. The process is followed by pitch-button polishing of the entire segment, to smooth the global surface and lower the raised edges. The maximum allowable mis-figure can be reached, but the average is more challenging.

The main reasons for this are as follows. First is print-through of edge-asymmetries from CNC grinding into the final result. The second reason relates to the pitch processing stage. If the process continues sufficiently to eliminate the edge and corner up-stands completely, it tends to disturb the global form and turn down the extreme edge. Furthermore, it can lead to a trench at the interface between the tool-lift zone and the bulk surface i.e. ineffective blending.

We report in this paper on different methodologies we have been researching on this issue, supported by theoretical modelling and experimental results. In particular, we report on bonnet ‘nodding,’ tool infill factor and local edge rectification.

## Methods

### Edge control with nodding bonnet tool

When a compliant bonnet-tool overlaps the edge of the part, excessive material is removed in the edge-zone due to increased local pressure. In ‘nodding’, as the IF moves along the tool-path and meets the edge of the part, the precess-angle is progressively increased. This maintains registration of the edge of the disk of polishing cloth with that of the part, truncating the IF, and avoiding edge-overlap (Fig. 1),

A bonnet for nodding needs first to be machined true to the machine virtual pivot (intersection of A, B axes), as is usual practice. The nominally circular disk of polishing cloth is molded to the bonnet radius and cemented to the bonnet. The periphery of the cloth is then machined, using a sharp tool, to be precisely circular and run true. This requires caution in order not to damage the bonnet. It is not necessary to machine right through the cloth; a step is sufficient to deliver an influence function with a sharp edge.

**Fig. 2:**
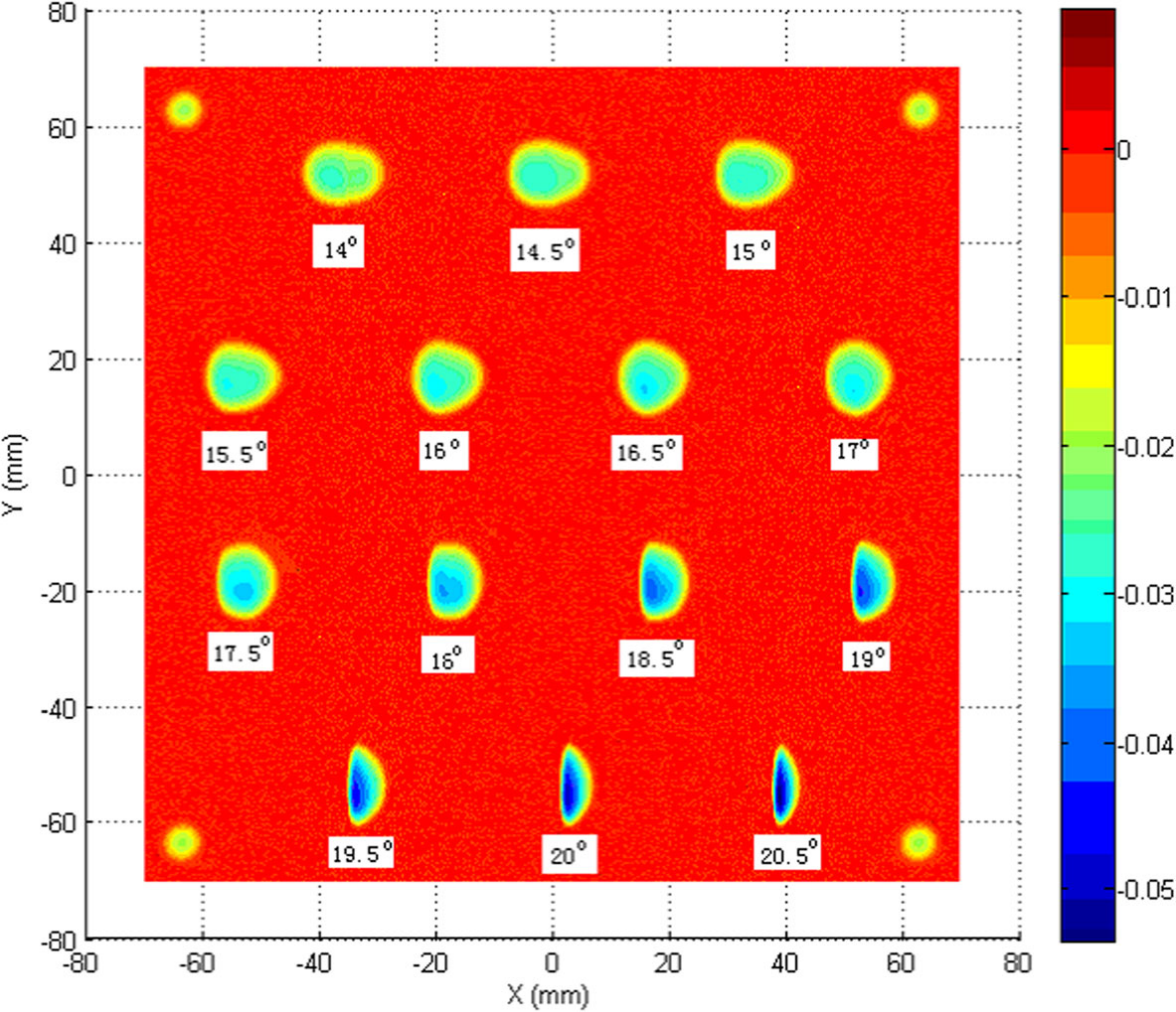
Generated influence functions with varied precession angle

Successful application of nodding for edge control relies on two aspects. First, a set of IFs in the edge zone requires stability and accuracy, including both removal rate and shape. Second, a nodding motion is required such that the truncated edge of the influence function is tangential to the edge of the part under polishing, as shown in Fig. 2.

**Fig. 3:**
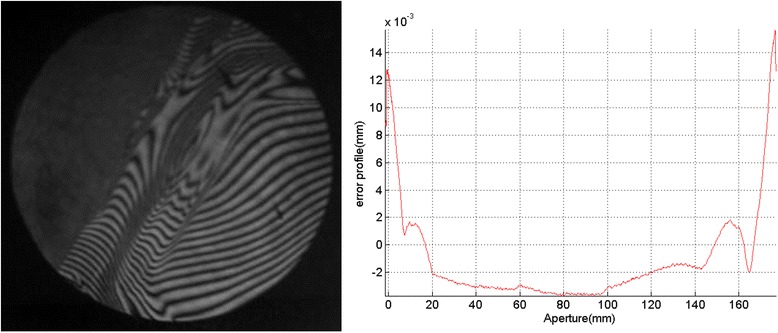
Processed surface edge with nodding method

**Fig. 4:**
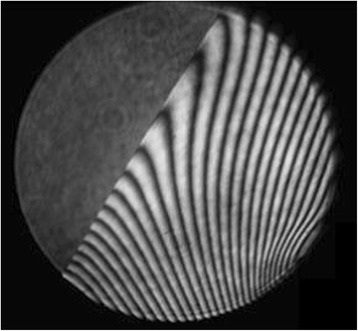
Sharp edge is obtained with pitch polishing after nodding process

An interferogram and Form Talysurf scan (edge to edge) of a surface processed with nodding, are shown in Fig. 3. It can be seen that a feature is left on the surface throughout the nodding zone. However, there is no edge turn-down. This demonstrates that the process is fundamentally sound. The up-standing edge is about 7 mm wide. This can be flattened by a pitch polishing process, as shown in Fig. 4.

### Edge control with non-uniform treatment-tools with different infill factors

Complementing bonnets, rigid tools can be used to rectify mid-spatial features and assist edge control. Their size is limited by aspheric misfit, leading to compliant (visco-elastic or non-Newtonian) materials often being incorporated. Traditionally, tools either ‘float’ on the part by gravity, or under additional spring-force. It is fundamentally not possible to achieve uniform removal throughout the bulk and edge zones. This would require the tool-path and tool to leave the part completely; impossible, because the tool would rock on the edge. Stopping the tool-path short leaves an excess of material, which cannot be fully rectified simply by changing process variables. This is exacerbated by other local boundary-condition issues, such as differences in slurry mobility on edges and bulk. To explore some of these effects, simulation work has been carried out to optimize process parameters to achieve optimal edge form.

**Fig. 5:**
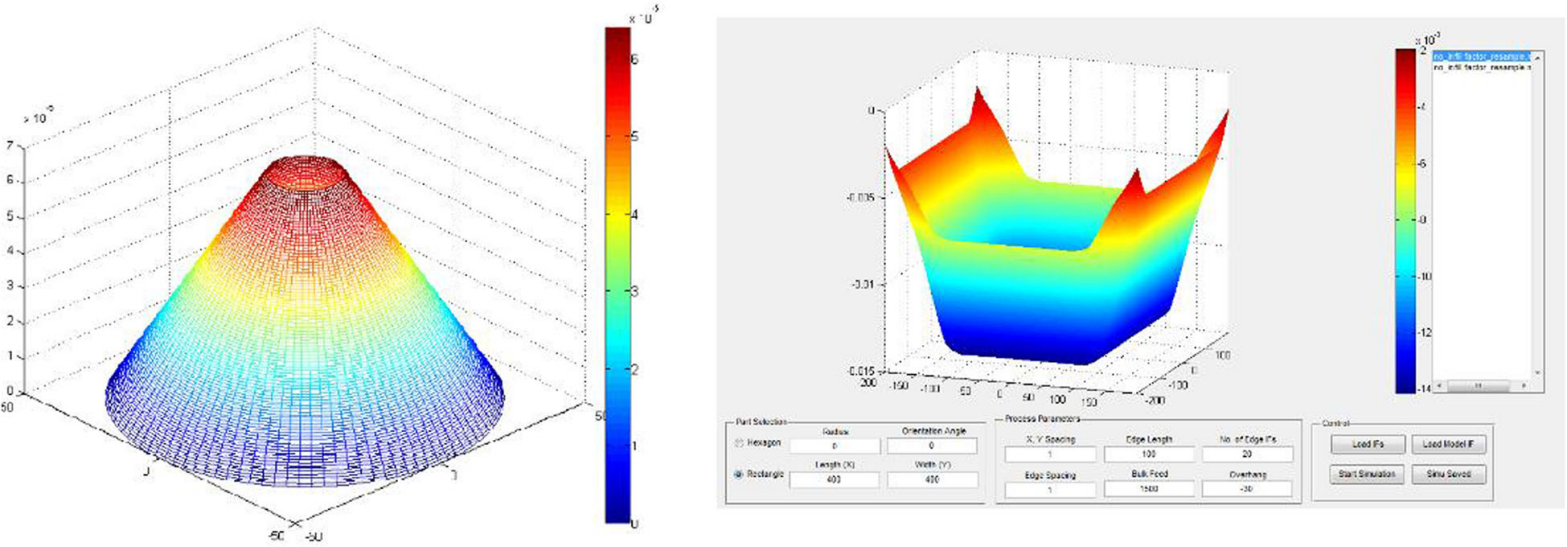
An example of tool influence function (**a**) and the surface profile modelling result is shown in (**b**)

A simulation has been conducted in the MatLab environment to predict the surface profile with rigid tool working on a flat workpiece surface based on Preston’s Law. The influence function of the rotating tool is computed (Fig. 5a) and the material removals then integrated over the tool path (Fig. 5b).

A variable we haven’t explored previously is the tool ‘in-fill factor’, applied to a rotating rigid or semi-rigid tool. We define this as the proportion of the circumference of each radial zone from tool-centre to tool-edge, which is occupied by a physical pad. In craft polishing this is represented by ‘petal laps’. To improve the relative removal ability on the edge, the infill factor on the edge of the part is fixed to be 1 and reduced as it comes closer to the centre, followed by Eq. 1:

1 $$ Infill Factor={\left(\frac{r}{R}\right)}^n $$

where R is the radius of the tool, r is the distance of concentric circles to the centre of the tool and n is the infill factor power of the equation.

**Fig. 6:**
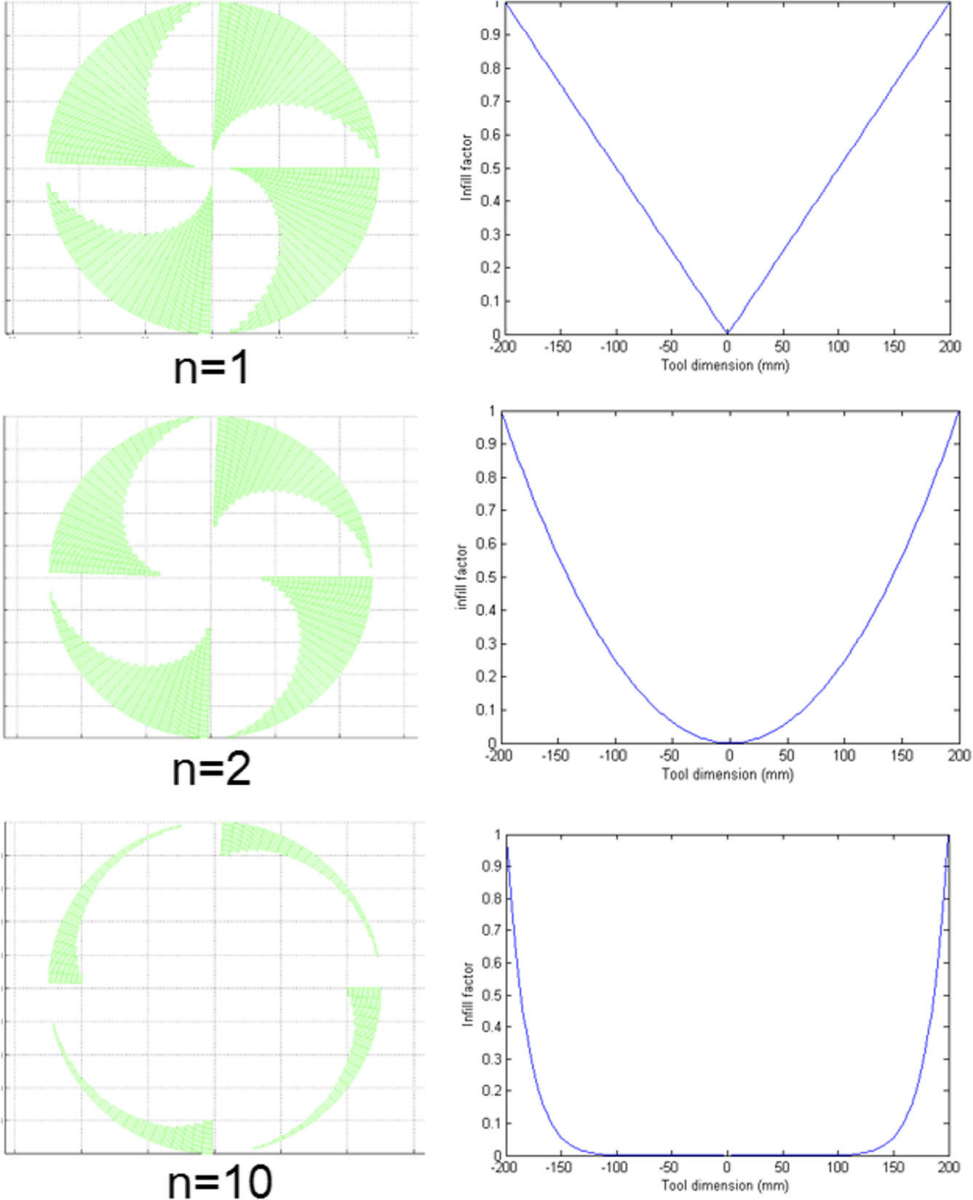
Some modelled tools modified by Eq. 1 with different infill factor powers

Some examples generated based on Eq. 1 with different powers have been modelled (shown in Fig. 6), where the coloured part represents the raised pad in contact with the part, and the white part the spaces between. It is expected that the tool with higher infill factor power can relatively remove more material on the edge than the bulk.

**Fig. 7:**
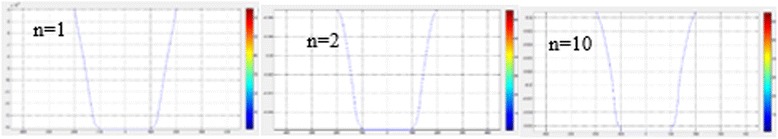
Cross sections of simulated surface profile after being processed by tools with different infill factors

The effect of such tools with different infill factor power has been simulated by integrating the corresponding influence function. Cross sections of the simulated surface profile is shown in Fig. 7. Interestingly, the modelling has shown that the edge-profile is *insensitive* to changing the in-fill factor in the manner shown, and therefore we have not progressed this idea further.

### Edge control with local rectification

**Fig. 8:**
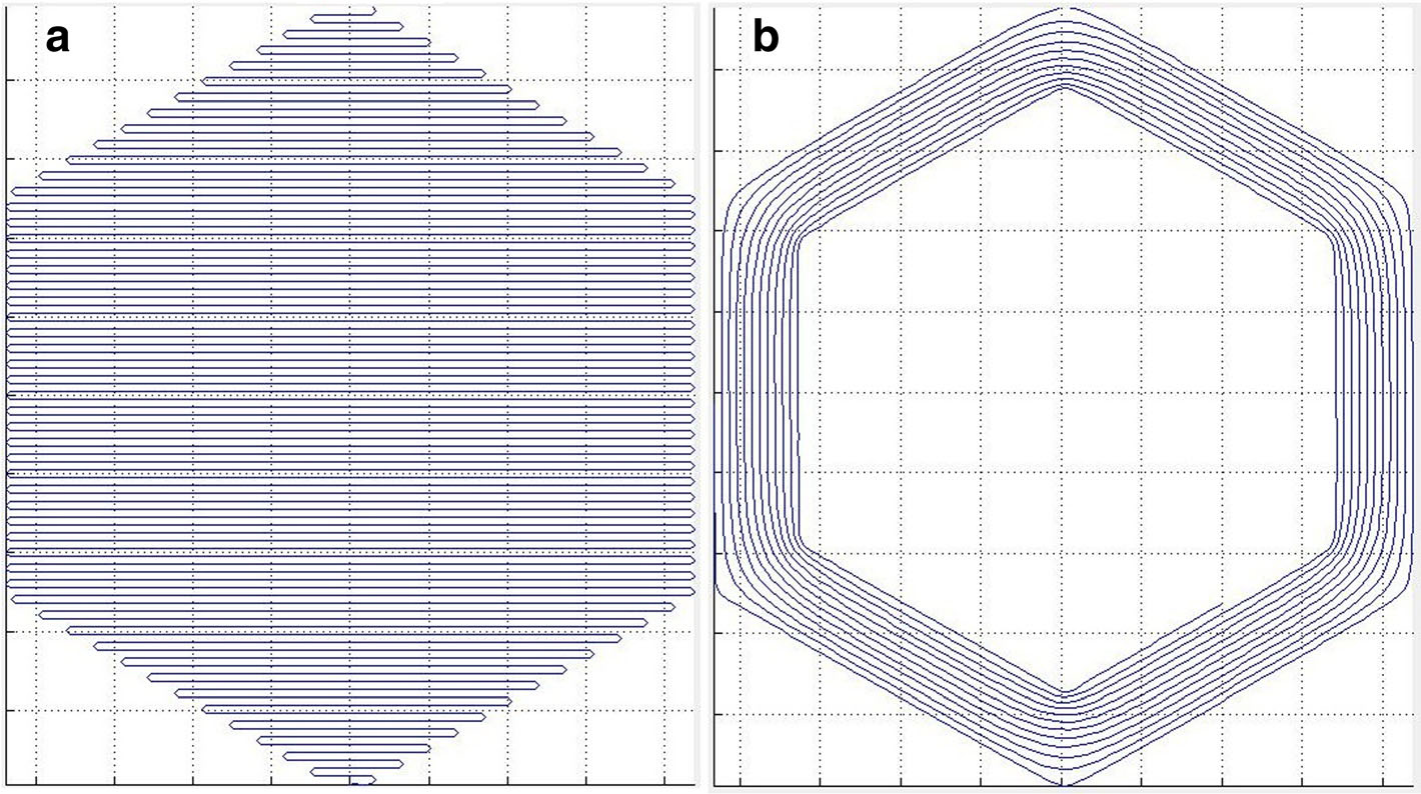
Schematic diagrams of tool paths (**a**) Raster tool path for bulk-surface. **b** Hex-Hex tool path for local-rectification (not to scale)

Local edge rectification means the polishing tool addresses the edge zones specifically, as shown in the illustrative hexagonal-spiral tool-path (Fig. 8). Preliminary work has been reported on modelling edge-zone correction using IFs generated where the polishing spot overlaps the edge. We report in this paper on experimental results, following development of a hexagonal tool-path, and software to model removal within the edge zone.

Correction of the edge-zone residuals may then proceed as follows. Bulk polishing is programmed to leave an upstanding edge-zone. An R20 bonnet is selected, as its small footprint gives greater sensitivity for local rectification than the larger bonnets used for bulk polishing. The R20 cores-out most of the up-stand using a hexagonal-spiral tool-path, constrained to lie within the edge-zone. Tool-lift is deployed towards the *inner* boundary of the edge-zone, to avoid creating a trench. Finally, a rotating pitch tool is used to ‘blend’ the edge zone into the bulk area.

Local rectification can improve edges of uneven height, and corners that are higher than the edges. Bonnet polishing tools used for the main bulk deliver spots too large for local correction at corners and edges. In principle, any bonnet can provide spot-sizes tapering to zero, but with larger bonnets the sensitivity to Z-offset errors becomes extreme. For example, assume a typical edge-correction spot-size of 6.9 mm. The corresponding Z-offsets for R20, R80 and R160 bonnets are 0.3, 0.07, 0.037 mm respectively. An error (increase) of a nominal 0.1 mm in Z offset then increases the area of each of the spots by 31%, 127% and 266% respectively. On the other hand, the process time will be unacceptable if the whole surface is processed with a small tool optimized for edges and corners.

#### Technical challenges and solutions

An error (increase) of a nominal 0.1 mm in Z offset then increases the area of each of the spots by 31%, 127% and 266% respectively. On the other hand, the process time will be unacceptable if the whole surface is processed with a small tool optimized for edges and corners.

The input quality for a local rectification step is a part such as a hexagon i) with the bulk form to specification, and ii) with unacceptable edge residuals that are all upstanding with respect to the extrapolated bulk form. The required fidelity of the mapping of the metrology-data coordinate-frame onto the part’s surface, and onto the machine’s CNC coordinate-frame is ≤ ~ 100 μm; significantly more demanding than the ~200 μm for the prior corrective polishing operation. This demonstrates one of the key advantages of performing such corrections, with on-machine metrology, on a dynamically-stiff Cartesian CNC platform of the quality of the Zeeko machines. A robot platform would hardly be competitive.

In the following section, various issues are discussed relating to metrology and polishing. The solutions provided have been experimentally verified.

##### Overshooting

When polishing and correcting bulk-form by dwell-time moderation, a significant offset or pedestal is required for two reasons i) to remove the overall surface and sub-surface damage layer from prior grinding, and ii) to avoid the infinite traverse speeds and accelerations that would be needed to ‘skip over’ the surface to allow zero local removal. Corrective polishing is then *differential* i.e. proportional with respect to the offset. Drifts in removal rate will affect the correction, but the principle of leaving edges always turned up provides a contingency.

For local edge-rectification of the up-stand, the material removal has to be controlled *absolutely*, so that the correction never overshoots i.e. it never causes the local surface to project below the extrapolated bulk-form, as this can be rectified only by re-working the entire *global* surface. Upward residuals, in contrast, can always be rectified with another *local* pass. There are several factors that can lead to overshoot, described below.

In the Zeeko process, the bonnet tooling is advanced towards the part prior to polishing, and first-contact is determined using feedback from a load cell in the polishing head. This gives the zero datum of bonnet compression. The tool is then advanced further towards the part (‘Z-offset’) to compress the bonnet, and so create a contact-spot (influence function) of the desired size. Any error in establishing first-contact directly disturbs the spot-size, and so affects volumetric removal rate by approximately a square law (see Section 3). This effect is therefore very sensitive, and is dominant in overshoot. It can be caused by movement of the part in its fixturing, thermal growth in the machine, residual hysteresis in the bonnet material, electrical noise in the load cell signal, or drift in the DC signal amplifier. The principal impact is an error in polishing-rate *between* acquiring influence function data and starting polishing.

**Fig. 9:**
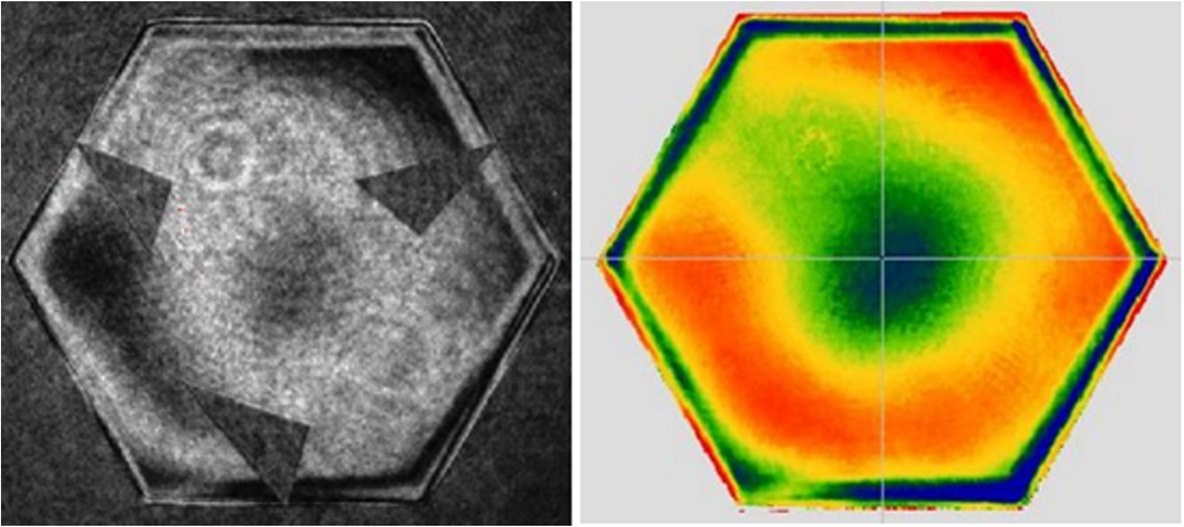
Overshoot due to touch-on error

Figure 9 gives a typical example, where such errors have led to excessive removal, creating a depressed trench inboard of the edge, of 270 nm in depth. The triangular masks visible in the interferogram are to identify the true physical edges (start of bevel), rather than the end of the visible fringes.

The solution to touch-on errors has been independent calibration, both immediately prior to acquiring influence function data, and immediately before polishing. The procedure is to perform touch-on, then back-off the tool through a known distance under software control, and manually check the distance by inserting and withdrawing a shim of known thickness. Any discrepancies can then be compensated in software through modifying the Z-offset.

A second cause of overshoot results from the definition of the depth-of-error to be removed. As mentioned above, skipping over areas is precluded by machine acceleration/speed limits. Thus, if an attempt is made fully to correct the edge-zone error, this will result in overshoot somewhere. This risk is mitigated by invoking the ability to change spot-size ‘on the fly’ in the *Precessions* process, by varying Z-offset. However, this is most sensitive to error when Z-offset and spot-size both approach zero i.e. around the area(s) of zero target removal. Therefore, a small positive residual is deliberately retained as a contingency, defined to be within the edge mis-figure specification.

A third cause of overshoot arises from any tilt of the part in the machine coordinate frame. The Z-offset variation will then be most at the edge zones, where the effect on volumetric removal rate is greatest. The standard Zeeko ‘Non Linear Correction’ algorithm performs touch-on at various locations over the surface, and the result of a numerical fit automatically corrects the CNC file. This in turn corrects the Z-offset along the tool-path, for any distortions or global tilts of the part on the polishing support system. However, the numerical fit is weighted in favour of the bulk area, by virtue of the predominance of sample points therein, and this can lead to errors in the edge-zone. A modified Non Linear Correction has been implemented, where samples are acquired only around the periphery of the part.

An additional factor is variation in specific gravity, temperature and/or pH of the slurry, between acquiring influence functions and polishing the part. On the IRP1600 machine used for full-size segment fabrication, this has been mitigated by increasing the slurry-tank volume to 150 l, increasing the slurry-delivery to the part to 30 l/min, and improving slurry agitation. Digital monitoring and archiving of slurry conditions has also been implemented.

##### Process time

**Fig. 10:**
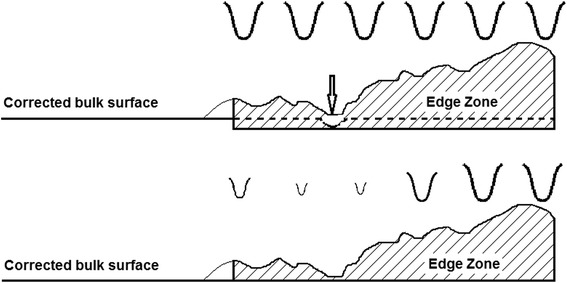
Principle of reducing process time. (Upper) Using uniform IFs requires a larger amount of material removal. (Lower) Deploy different size of IFs requires fewer material removal

The aim of the local edge-rectification is to reduce both the residual edge mis-figure and the total process time, with respect to the standard process-chain that rasters the entire surface. In this standard process, the pitch-button time is dominated by the need to lower the upstanding edges. However, this also removes an unnecessarily large DC level from the polished bulk area, and global form tends to regress, as shown in Fig. 10 (upper). With local rectification, the volume of the edge up-stand is reduced by typically 80% using a small bonnet/spot-size, operating *only* in the limited area around the edge (Fig. 10 lower). This in turn increases the effectiveness, and reduces the time, for the final pitch-button step. On the experimental sample, the total process time saved is 165 min which is 22% of the total process time. On a real segment, this saving would increase due to fact that the time spent on the bulk area will increase by a square law, whereas the time increase on edges will be of linear.

##### Optimising the bulk surface polishing for edges

The first process step is to pre-polish the surface with a large polishing tool. This is to remove subsurface damage left by the prior generating process and to improve surface roughness so that global form can be measured by full-aperture interferometry. The polishing tool in the work on witness parts reported here is an R200 bonnet, with 2.8 mm Z-offset, delivering a nominal 60 mm diameter polishing spot. The target removal was 10 μm P-V depth. Edge control at this stage aimed to leave an up-stand, so that this can be locally corrected afterwards. Accurate metrology of the edge profile is very important in debugging the process, and so a fiducial was engraved on the surface of the witness-part surface to act as:-

**Fig. 11:**
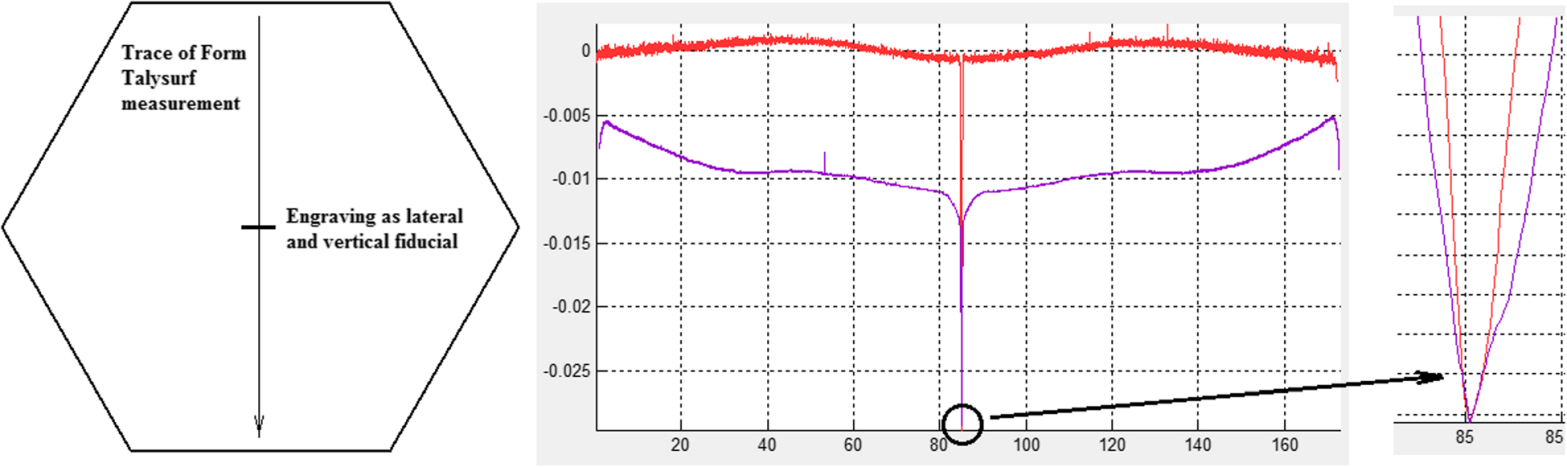
Selection of fiducial when using profilometer

1. A datum to measure absolute material-removal by comparing surface profile measurements across the engraved mark before and after polishing, shown in Fig. 11.
2. A fiducial to register the lateral position of profile measurements so that true edge positions were known.

**Fig. 12:**
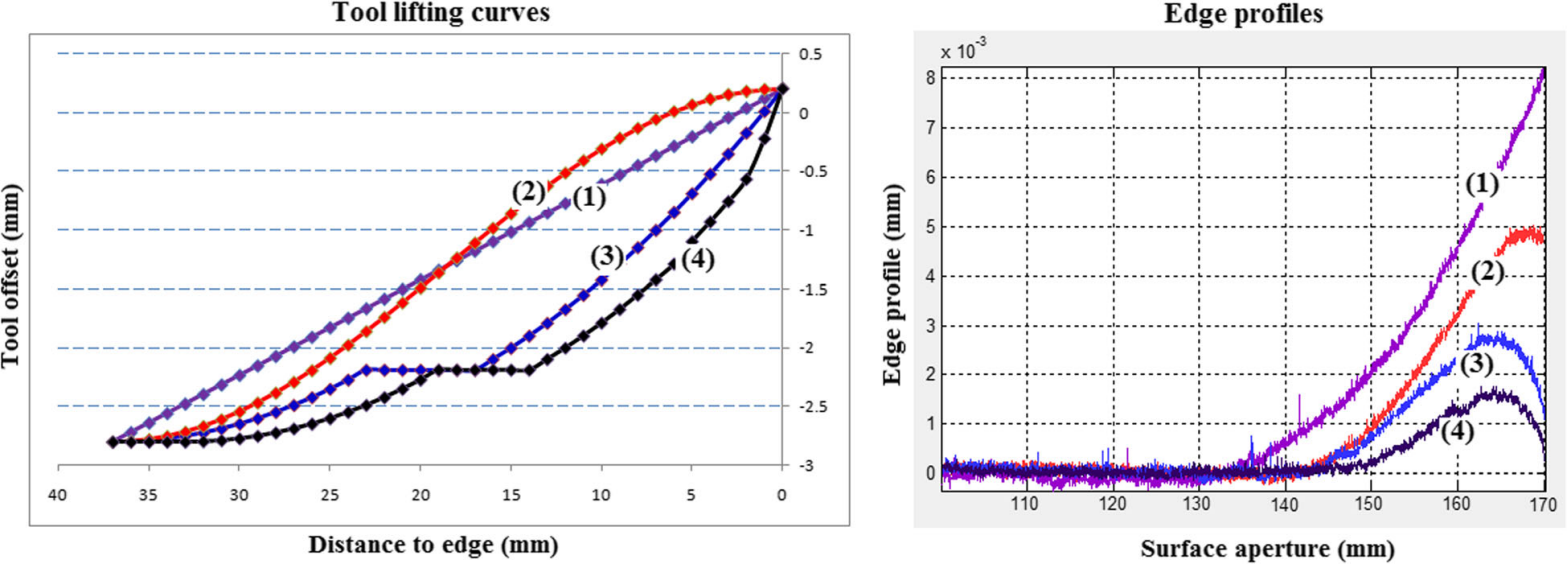
Tool-lift curves (left) and resulting edge profiles (right)

Figure 12 (left) shows examples of several tool lifting schemes we have investigated. The horizontal axis represents the distance from the centre of the polishing spot, to the extreme edge of the part. The vertical axis comprises the Z-offsets on the surface with respect to touch-on. Different tool lifting parameters have produced different edge profiles, as shown in Fig. 12 (right), where the extreme right of the graph represents the true edge of the surface.

There are several parameters that can be explored, such as precess-angle, local dwell time, Z-offset, and maximum overhang of the polishing spot at the edge. Typical process parameters are listed below in Table 1. With these parameters, a total of 10 μm can be removed through 3 polishing runs. The polishing should start from every other corner to create a symmetric up-stand.

**Table 1:** Process parameters of large tool polishing

Parameter	Value	Unit
Surface feed	2000	mm/min
Head speed	800	RPM
Precess angle	14	Degree
Tool offset	2.8	mm
Tool overhang	0	mm
Track spacing	3	mm

##### Mathematical basis of hexagonal spirals

We have developed two new forms of spiral tool-path; starting from the original circular-spiral, to the ‘adaptive spiral’ and ‘hex-hex’ spiral, which are reported below.

The adaptive spiral starts by following the edge of the part (e.g. hexagonal), and morphs progressively to a circular spiral at the centre. The hex-hex spiral does not morph, but stays (e.g.) hexagonal between the outer boundary of the part, and a defined inner boundary on the surface. In both cases, if the traverse were slowed to zero at the corner, then accelerated to follow the direction of the new edge, a depression at the corner would result. The corners must therefore be rounded.

The adaptive spiral has previously been successfully applied to cutting process such as high speed routing http://www.wseas.us/e-library/transactions/mechanics/2008/27-159.pdf and 5-axis milling http://www.sciencedirect.com/science/article/pii/S0168927403000394. One method to compute adaptive spirals consists of computing the solution to Poisson’s equation:

2 $$ -\boldsymbol{\operatorname{div}}\left(\boldsymbol{\operatorname{grad}}\left(\boldsymbol{u}\right)\right)=1 $$

with Dirichlet conditions applied to the boundaries of the surface:

3 $$ \left\{\begin{array}{c}\ \boldsymbol{u}=0\kern1em \boldsymbol{for}\ \boldsymbol{outer}\boldsymbol{boundaries}\\ {}\ \boldsymbol{u}=1\kern1em \boldsymbol{for}\ \boldsymbol{inner}\boldsymbol{boundaries}\end{array}\right. $$

**Fig. 13:**
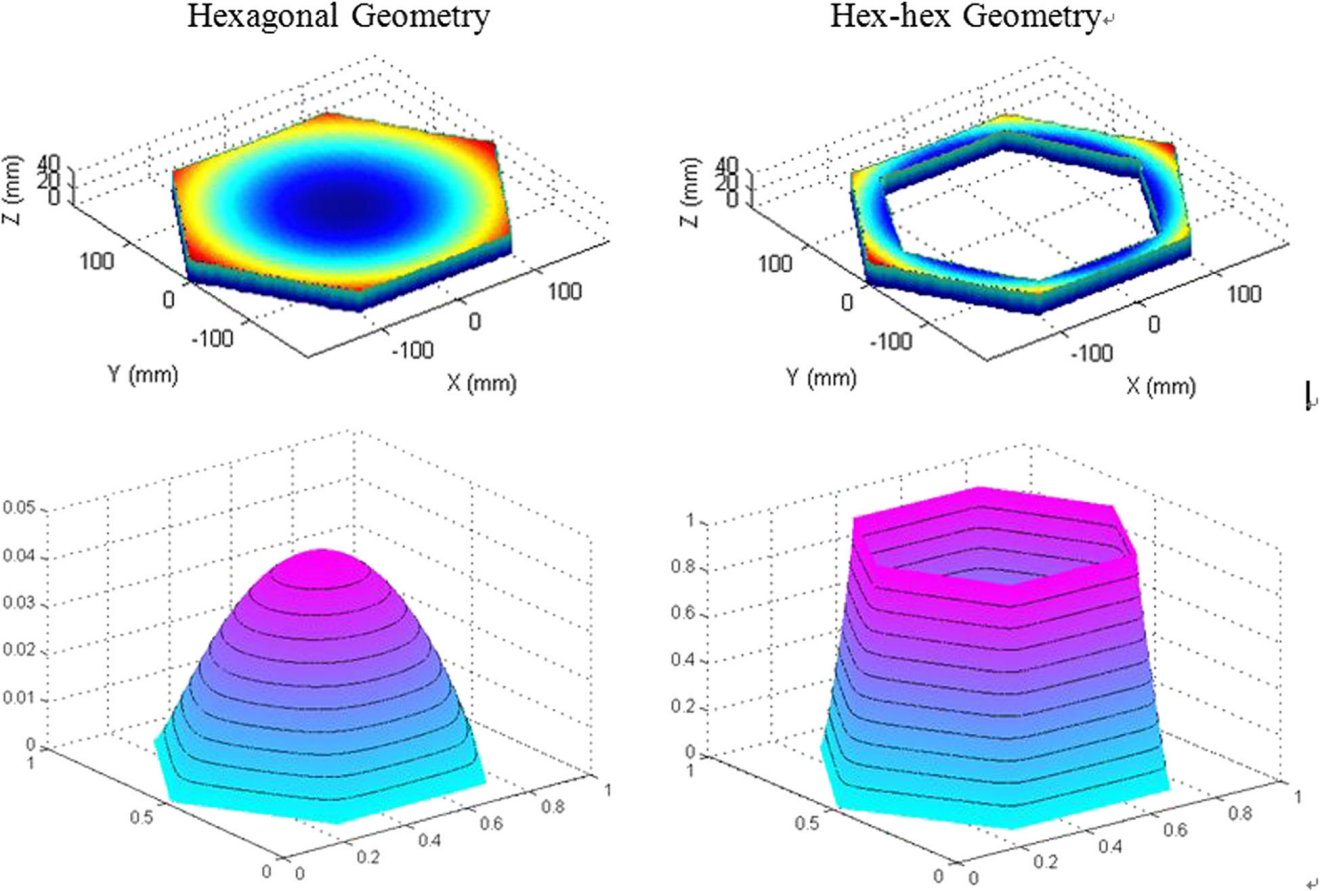
Geometry (top), solution of Poisson’s equation with iso-contours (bottom)

A hex-to-hex surface (upper right) and associated solution of Poisson’s equation (bottom left) are shown in Fig. 13. The tool path is then generated by following iso-contours on the solution to Poisson’s equation (bottom right).

**Fig. 14:**
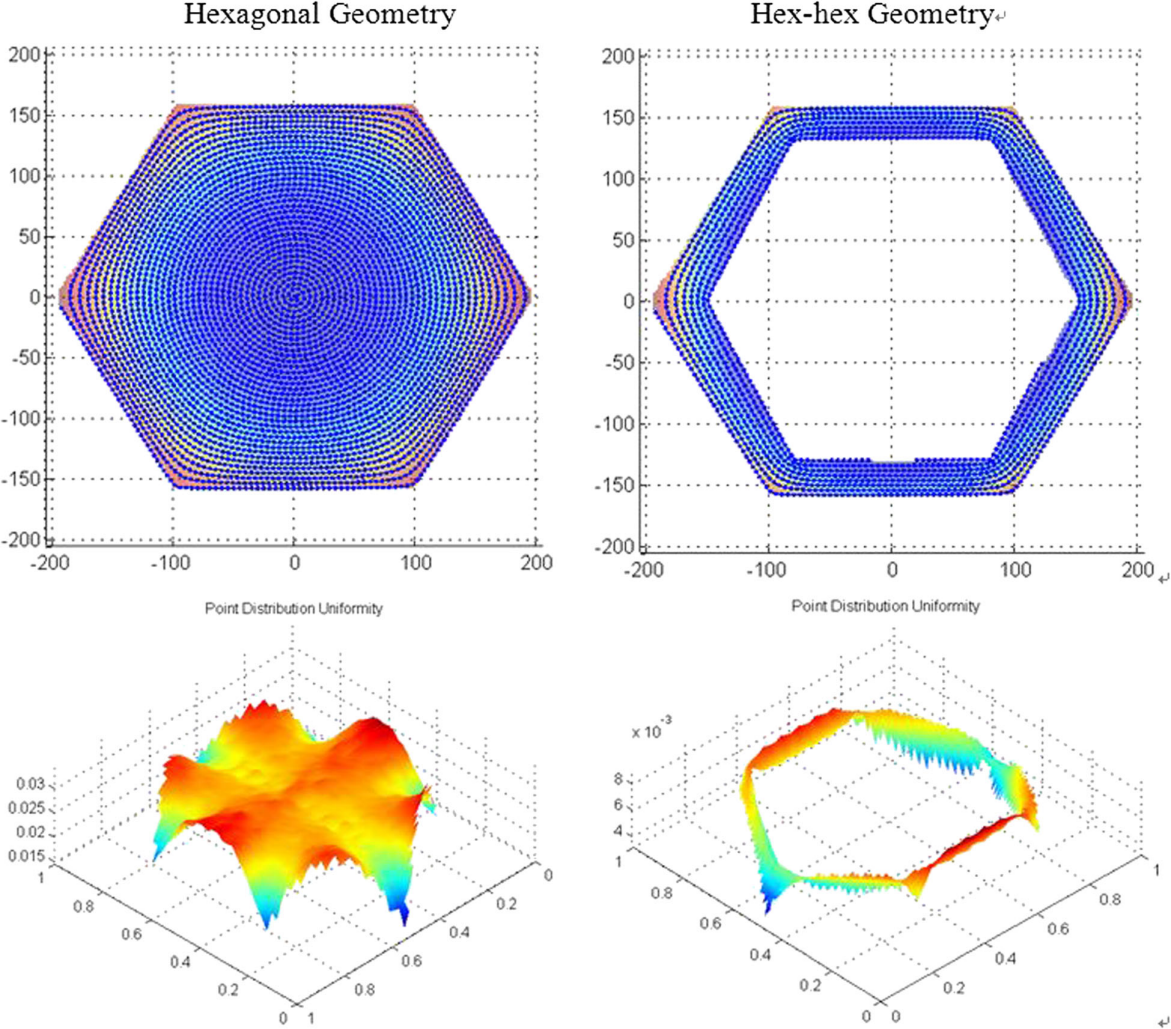
Tool paths (top) with their associated point distribution uniformity (bottom)

While this method can generate hex-hex and adaptive tool paths featuring smooth cornering, a drawback is that the track-spacing changes progressively across the tool path. Since bonnet polishing is a time and space dependent sub-aperture process, a change in track-spacing introduces local variations in removal depth. To compensate for this effect, it is necessary to compute the relative density of tool-path points across the surface, and use this information to moderate the feed rate of the polishing spot along the tool path. This density can be computed by convoluting the dataset with a Gaussian filter:

4 $$ \boldsymbol{g}\left(\boldsymbol{x},\boldsymbol{y}\right)=\frac{1}{2\uppi {\upsigma}^2}\bullet {\boldsymbol{e}}^{-\frac{{\boldsymbol{x}}^2+{\boldsymbol{y}}^2}{2{\upsigma}^2}} $$

where σ relates to the width of the spot size used during subsequent polishing. Examples of tool paths with their resulting point distribution uniformity are shown in Fig. 14.

Finally, it is possible to balance tool path smoothness against the uniformity of point distribution by introducing extra coefficients in Poisson’s equation:-

5 $$ -\boldsymbol{\operatorname{div}}\left(\boldsymbol{coef}{1}^{\ast}\boldsymbol{\operatorname{grad}}\left(\boldsymbol{u}\right)\right)+\boldsymbol{coef}{2}^{\ast}\boldsymbol{u}=\boldsymbol{coef}3 $$

**Fig. 15:**
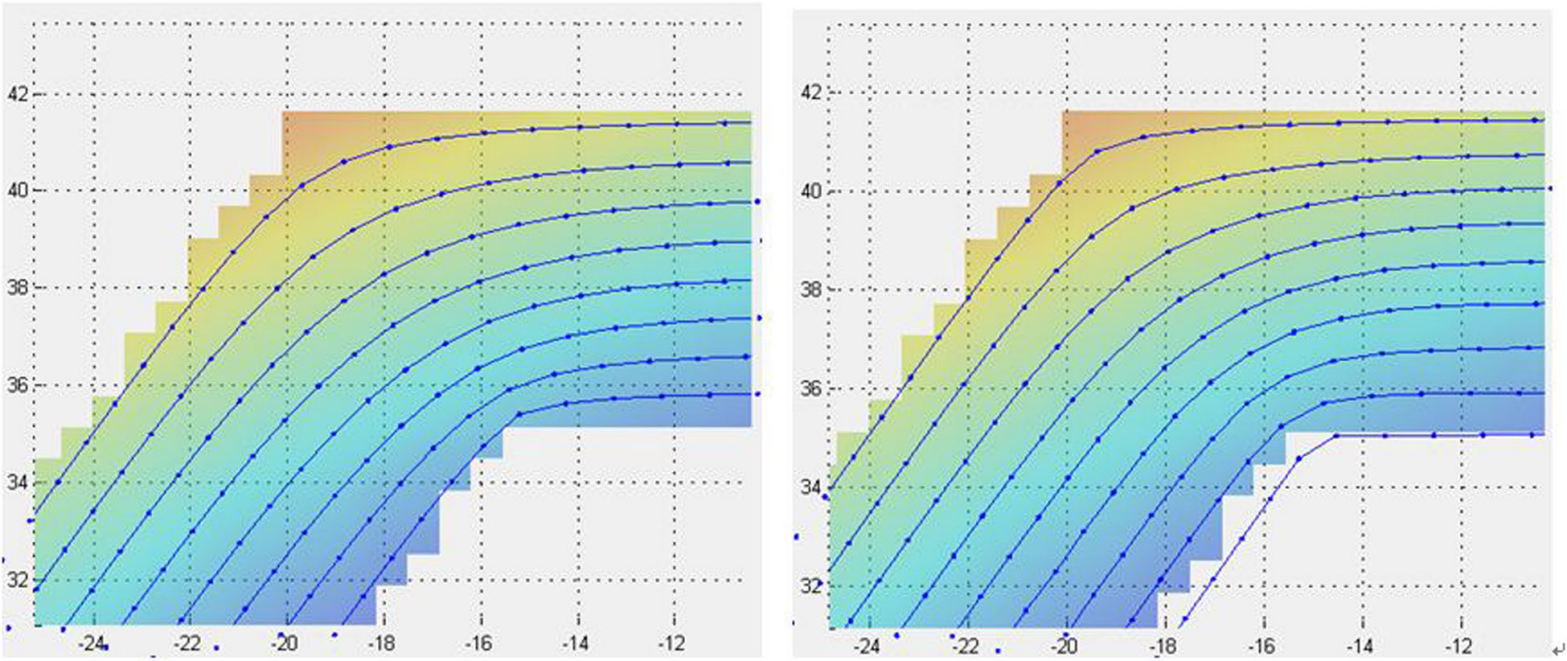
Corner area of tool paths calculated with different sets of coefficients

The corner area of tool paths calculated for different coefficients on the same hex-hex surface are shown in Fig. 15.

## Results and discussion

Process optimization was carried out on a Zeeko IRP1200 machine. The demonstration part used was a borosilicate hexagon of 390 mm corner-to-corner. The surface was spherical with a radius of curvature 3.0 m for ease of testing. An interferometer was located on a test tower above the Zeeko polishing machine to provide in-situ metrology.

**Fig. 16:**
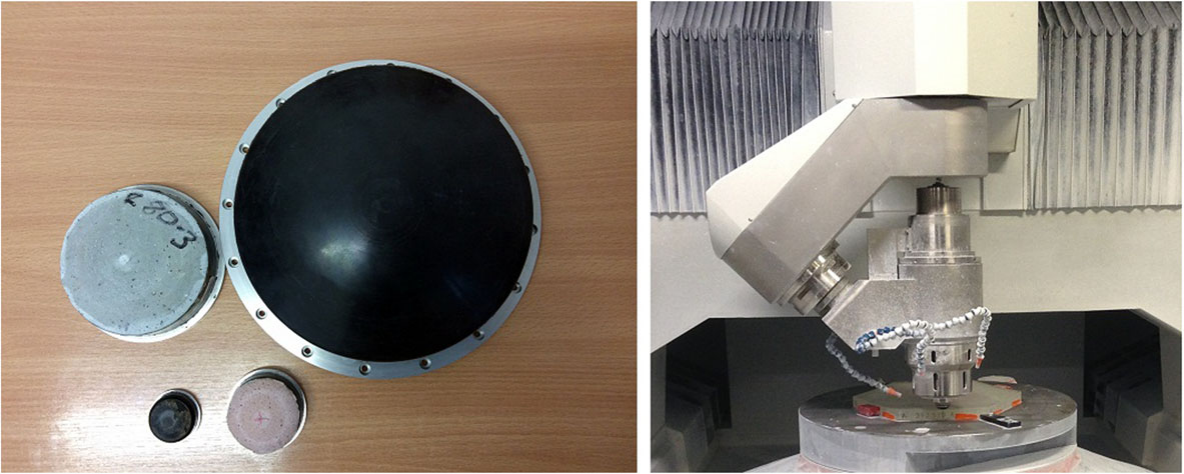
(**a**) (left) Bonnets used (R200, R80, R40 and R20) (**b**) (right) IRP1200 machine in probing mode

The part was pre-polished using an R200 bonnet shown in Fig. 16 (a). This removed subsurface damage from generating the spherical form, and improved surface quality so that full-aperture interferometry could be deployed. Smaller tools, such as R80, were then used for form-correction and during these stages, process parameters were optimized for minimum edge-zone up-stand. Local edge rectification then used a R20 bonnet, delivering spot-sizes up to 6.9 mm. These small spot sizes were well-matched to the spatial frequencies in the up-stand. The use of Uninap polishing cloth from Universal Photonics provided a benign removal characteristic contributing to the precision of removal.

**Fig. 17:**
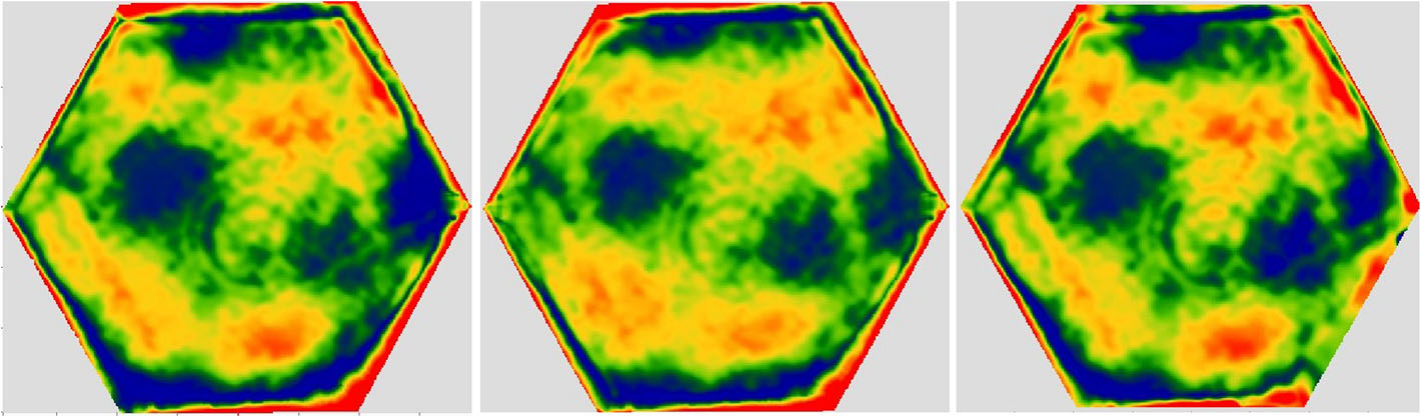
**a** Interferometry after form corrections. **b** After1^st^ and (**c**) 2nd local edge rectification. The average edge mis-figures after the 3 stages were 108 nm, 91 nm and 72 nm respectively

The surface after form correction is shown in Fig. 17 (a). The errors of the entire surface were 27 nm RMS and 182 nm PVq (95%). The average edge zone mis-figure was 108 nm PVq. For the first local edge rectification, 30% of the total edge error was targeted and the polishing time was 49 min. The average error mis-figure was 91 nm after this correction, as can be seen in Fig. 17 (b). The second run target 70% of the total error in edge zone. This was to avoid over-polishing of certain areas due to residual polishing spot-variation, even after surface tilt compensation. The average error mis-figure was 72 nm after this run, as can be seen in Fig. 17 (c).

**Fig. 18:**
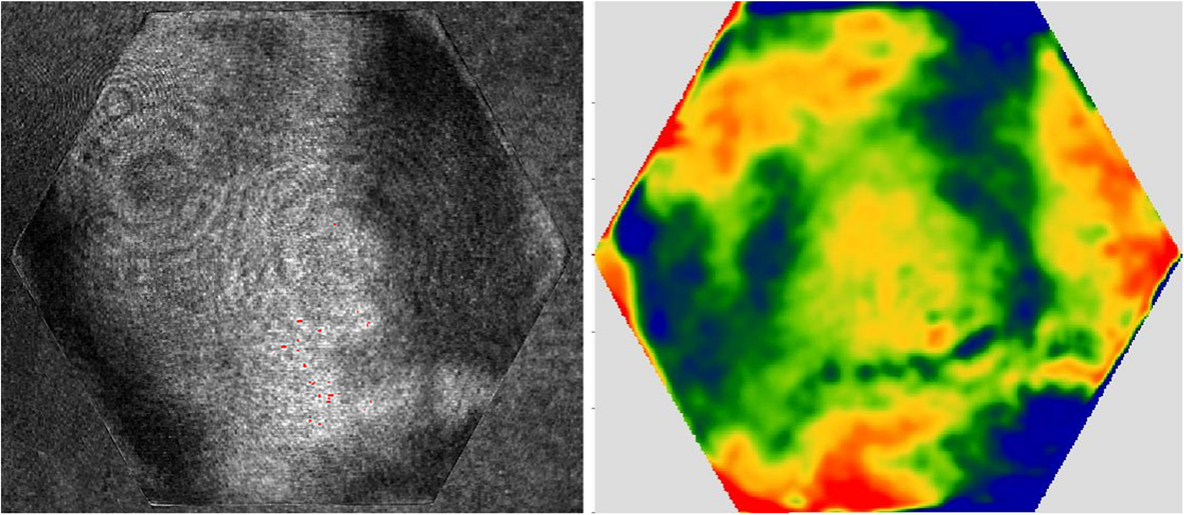
Surface fringes and phase map after final blending

The final process was blending of the edge zone into the bulk area using a pitch-button tool. The diameter of 50 mm was selected for compatibility with processing a full-size segment, to meet the criterion that the aspheric misfit would be well below the slurry particle size [11]. This step also helped to remove certain mid-spatial frequency features resulting from small-tool corrections. The edge mis-figure after this process, averaged over the six individual PVqs, was 68 nm PVq, as shown in Fig. 18.

## Conclusions

We have previously reported on our edge-control work, in regard to a process-chain that operates on the entire segment surface using raster tool-paths. This has resulted in a full-size segment being completed to specification. In this paper, we report on a new extension to the process, where we have treated the edge-zone separately with small tools, without disturbing the finished bulk area. We have pointed out that this requires the edge-zone residuals to be high with respect to the extrapolated bulk surface, which the tool-lift method can deliver.

We have identified specific issues related to successful local edge rectification, and reported on our simulation and tool-path software-development. In particular, simulation has demonstrated that superior edge-quality can be achieved with small-tool local rectification, compared with processing of the bulk surface. Furthermore, constraining the tool-path to follow the edge of the part drastically reduces rectification time compared with using the same tool to raster-polish the entire surface. New software functions have been developed to execute a hexagonal tool path, modified in the corners to accommodate acceleration/ deceleration limits of the machine. Modified probing software has also been implemented, so that probing is constrained to the edge-zone, in order to improve precision of determining tip/tilt of the part.

Further work to be conducted will focus on understanding the trade-off between edge-quality and total process-time. We have confirmed that edge-quality improves with smaller tool-sizes, and that total process time is reduced if rectification is constrained to the edge-zone alone. If tools smaller than R20 are invoked for finer edge rectification, total process-time will start to increase. Nevertheless, this may confer advantages in some applications if more stringent edge-specifications have to be met.

## Abbreviations

CNC: Computer numerically controlled
E-ELT: European extremely large telescope
IF: Influence function
PV: Peak-to-valley
